# Triple network model of brain connectivity changes related to adverse mood effects in an oral contraceptive placebo-controlled trial

**DOI:** 10.1038/s41398-023-02470-x

**Published:** 2023-06-16

**Authors:** Esmeralda Hidalgo-Lopez, Jonas Engman, Inger Sundström Poromaa, Malin Gingnell, Belinda Pletzer

**Affiliations:** 1 Department of Psychology, University of Salzburg, Salzburg, Austria; 2 Centre for Cognitive Neuroscience, University of Salzburg, Salzburg, Austria; 3 Department of Psychology, University of Michigan, Ann Arbor, MI USA; 4 Department of Anesthesiology, Chronic Pain and Fatigue Research Center, University of Michigan, Ann Arbor, MI USA; 5 Department of Psychology, Uppsala University, 751 85 Uppsala, Sweden; 6 Department of Women’s and Children’s Health, Uppsala University, 751 85 Uppsala, Sweden; 7 Centre for Women’s Mental Health during the Reproductive Lifespan, Uppsala University, 751 85 Uppsala, Sweden; 8 Department of Medical Sciences, Uppsala University, 751 85 Uppsala, Sweden

**Keywords:** Neuroscience, Psychiatric disorders, Biomarkers

## Abstract

Combined oral contraceptives (COC) are among the most commonly used contraceptive methods worldwide, and mood side effects are the major reason for discontinuation of treatment. We here investigate the directed connectivity patterns associated with the mood side effects of an androgenic COC in a double-blind randomized, placebo-controlled trial in women with a history of affective COC side effects (*n* = 34). We used spectral dynamic causal modeling on a triple network model consisting of the default mode network (DMN), salience network (SN) and executive control network (ECN). Within this framework, we assessed the treatment-related changes in directed connectivity associated with adverse mood side effects. Overall, during COC use, we found a pattern of enhanced connectivity within the DMN and decreased connectivity within the ECN. The dorsal anterior cingulate cortex (SN) mediates an increased recruitment of the DMN by the ECN during treatment. Mood lability was the most prominent COC-induced symptom and also arose as the side effect most consistently related to connectivity changes. Connections that were related to increased mood lability showed increased connectivity during COC treatment, while connections that were related to decreased mood lability showed decreased connectivity during COC treatment. Among these, the connections with the highest effect size could also predict the participants’ treatment group above chance.

## Introduction

Combined oral contraceptives (COC) are among the most commonly used contraceptive methods worldwide [1]. First introduced in the 1960’s, nowadays more than 151 million women of reproductive age use this method, and more than 80% of women from the US have used it at some point in their life [2]. COCs contain a synthetic estrogen, commonly ethinyl-estradiol, and a progestin that can be classified as androgenic or anti-androgenic, depending on its interaction with the androgen receptor [3, 4]. These exogenous hormones exert, via negative feedback, downregulation of the hypothalamic–pituitary–gonadal axis, and therefore, decrease the endogenous ovarian hormone production. Although research is still scarce, the continued exposure to synthetic progestins, has been proposed to underlie the adverse mood effects that some women experience during COC treatment [5, 6]. While observational studies come to different conclusions as to the risk of developing affective disorders [6, 7], mood side effects are the major reason for discontinuation of COC treatment [8, 9]. Randomized, placebo-controlled trials (RCTs) on adverse mood symptoms are rare, but the prevalence of this side effect is estimated to be between 4–10%, particularly for androgenic COC [9]. In a well-powered RCT, Zethraeus et al. [10] tested the effect of an androgenic COC (levonorgestrel), which decreased vitality, well-being and self-control compared to placebo. Lundin et al. [5] carried out an RCT with a partially anti-androgenic COC (nomegestrolacetate), with premenstrual improvement in depression but similar adverse inter-menstrual mood-related effects, such as enhanced irritability and mood lability, compared to placebo. At the same time, anti-androgenic COCs may have beneficial effects on mood, especially for women with pre-menstrual symptoms [11]. Thus, the susceptibility for adverse mood effects during COC treatment varies between subjects. However, up to now little is known about individual risk factors.

Although mood-related side effects are usually more pronounced in women with a history of depressive symptoms, depressed mood is not consistently reported to change after treatment in RCTs [10, 12]. These depressive symptoms may, however, appear in the long term, given that prospective cohort studies have associated the use of COC (and hormonal contraception in general) with the subsequent depression diagnosis or use of antidepressants [6]. It has been suggested that women who experience adverse mood-related side effects during COC use, show alterations in emotional processing, including impaired emotion discrimination, reactivity, and response (see review [13]). For instance, deficient fear extinction [14], impaired emotion recognition [15, 16] or differential emotional memory [17, 18] have been reported in COC users. These processes are related to the symptomatology of mood disorders and have also been related to differences in large-scale brain organization [19], i.e., intrinsic brain networks. Intrinsic connectivity networks are characterized by temporally correlated activity at rest, and can be identified by functional magnetic resonance imaging (fMRI). Previous neuroimaging studies have already found differences between COC users and naturally cycling women, and changes between the active and inactive phase of the pill in the functional connectivity of the brain at rest, either focusing on connectivity patterns of single brain areas, or intrinsic connectivity networks [20–26]. Differences in brain structure and function of individual brain areas between naturally cycling women and COC users have also been reported and related to emotional and cognitive processing (for a review, see [27]). Accordingly, it is of particular interest, whether women who experience adverse mood-related side effects during COC use show changes in large-scale brain organization and whether these changes can be linked to the severity of their mood symptoms.

Recent years have witnessed an increased integration of psychiatry and neurosciences, which allows us to change our perspective on mental well-being and further understand its underlying neural correlates. The triple network model proposes that a balanced connectivity between three ‘core’ networks, i.e., the default mode network (DMN), the salience network (SN) and the executive control network (ECN), is crucial for mental health [19]. These three networks support basic and higher-order regulatory functions fundamental to information processing and are thus essential to a variety of cognitive and emotional functions. The DMN is related to self-referential mental activity and increases its activity during the resting state. Its major nodes include the precuneus/posterior cingulate cortex (PCC), bilateral angular gyrus (AG) and medial prefrontal cortex (mPFC) [28]. The SN is important for detecting and integrating salient external and internal stimuli [29]. It is comprised by bilateral anterior insula (AI) and dorsal anterior cingulate cortex (dACC). The SN, especially the insular cortex, acts as a switcher between networks and has been characterized in healthy population as engaging the ECN in response to relevant stimuli [30]. The ECN is a frontoparietal network related to the active manipulation and inhibition of information and thus associated with planning, decision-making, and the control of attention and working memory [29]. It includes the bilateral middle frontal gyri (MFG) and bilateral supramarginal gyri (SMG). Deficits in dis/engagement between the nodes of these three networks are suggested to be particularly relevant for affective disorders [19]. With regards to emotional stimuli, this could lead to a weakened salience map and aberrant filtering of stimuli [19], and both depression, social anxiety and panic disorder have been associated with reduced connectivity within the SN, together with hyper-connectivity between the DMN (especially medial areas) and the SN [31]. For the ECN, hypo-activation has been found in frontal areas of both depressed and anxious patients, while hyper-connectivity between frontal nodes of the ECN and SN (MFG-ACC) has been suggested to reflect a compensatory mechanism [31].

Given the sparsity of literature, it is challenging to draw a general conclusion regarding the differences between COC users and naturally cycling women in resting state fMRI (for a review, see [32]). Some studies have reported differences within the intrinsic functional connectivity of the DMN [21, 26], the SN [20, 26] and the ECN [20, 21, 26], though results are not without inconsistencies [22]. However, these studies (i) were cross-sectional, which affects the statistical robustness, (ii) used different cycle phases in naturally cycling women as control group, (iii) or defined intrinsic connectivity networks according to different classifications, which makes comparisons between findings difficult. In a within-subject longitudinal design, functional changes between the active and inactive phases of the pill have been reported in the subcortical-DMN connectivity of the resting brain [33]. However, the scanning sessions of this study were preceded by exposure to a stressor, which may impact the networks under study. More recently, in a single case study, the brain network dynamics affected by the endogenous ovarian hormone fluctuations were shown to be supressed during the intake of COCs [24, 34]. A further graph-theoretical approach was taken to analyse the connectivity architecture of this data, showing that during COC use, brain organization was less segregated and modular than during the natural menstrual cycle [35].

Despite the advantages of functional connectivity, these approaches may be too coarse and insufficient to capture within and between network connectivity dynamics. In general, they do not capture changes in the large-scale organization of the brain, given that (i) functional connectivity measures do not provide information about the directionality of connectivity changes and/or (ii) intrinsic connectivity approaches are inadvertently restricted to changes in connectivity within a given network, while information processing requires the balanced integration of information across different networks. We here aim to disentangle the directed connectivity patterns particularly in women experiencing adverse mood side effects to an androgenic COC. Given that the triple network model has already been proved useful to investigate connectivity changes related to hormonal fluctuations [36], we propose it here as a valuable framework to study the COC-dependent modulation of connectivity patterns. To address this question, we used spectral dynamic causal modelling (DCM) to analyse data from a double-blind RCT, for which women were selected based on previous adverse mood effects on COC and where the COC group experienced increased mood lability, fatigue and depressed mood compared to the placebo group [12]. We assessed the differences between causal connectivity strength before and during treatment within a Hierarchical Bayesian framework [37]. This allowed us to capture the main effects of group (placebo vs. COC), and treatment (pre vs. during), and most relevant for our research question, the interaction of both factors [38].

Given that previous research hints at an enhanced connectivity within the DMN (especially medial areas), but decreased connectivity within the ECN related to depressive symptoms [31, 39], we expect this pattern of within-network connectivity also during COC treatment in comparison to placebo, and that the changes in the pattern should be related to depressive mood. Regarding between-network connectivity, functional connectivity findings from this dataset already show an increased connectivity between the medial nodes of the SN and DMN (dACC and PCC) during COC treatment [40], though the directionality of this effect remains unclear. Using DCM among the nodes of the triple network model, will allow us to resolve the directionality of this interaction and other interactions between the SN and DMN, and also explore the directed connectivity of these nodes to the ECN. Related to a potential impaired filtering of stimuli, we also expect a weaker engagement of the frontal ECN by the SN [19] and decreased afferent connectivity into the SN during COC treatment [41, 42].

## Materials and methods

### Participants

Thirty-five healthy women with previously reported COC-induced mood deterioration participated in a double-blinded, randomized, parallel-group clinical trial. The sample size of the present study was chosen based on previous studies revealing that an approximate number of 20 participants is sufficiently large to get robust model predictions [43] and previous research applying spectral DCM to resting state fMRI in a similar sample size [44, 45]. Exclusion criteria were use of hormonal contraceptives, cortisol, levothyroxine, or psychotropic drugs within the previous two months; and ongoing neurological or psychiatric disorders. The COC-induced mood deterioration was validated by a semi-structured interview. All participating women reported at least one symptom in relation to previous COC use, most commonly depressed mood (88,2%) and mood swings (82.4%), followed by irritability (70.6%), decreased interest in usual activities (44.1%), and/or anxiety (38.2%) [12]. Blinding, packing and randomization was done by Apoteket Production and Laboratories, Stockholm, Sweden. The participants were randomized to either a COC (ethinyl estradiol (EE) 30 mg/0.15 mg levonorgestrel, provided by Bayer Pharma AB) or placebo (Bayer Pharma AB), each given for one treatment cycle (21 days). One participant, randomized to placebo, discontinued the study before the second session. As previously reported, women in the COC and placebo groups did not differ in age, educational level, previous COC history and prevalence of self-reported pre-menstrual syndrome (PMS) symptoms (see Table 1). Regarding the last reported COC use, most had been taking COCs containing an androgenic progestin (29 women, 85.3%), while 5 participants had used COCs with anti-androgenic progestin. The time range since last COC intake was 2 to 108 months prior to randomisation (mean = 22.3, sd = 23.4). Results from task-based paradigms and salience resting-state functional connectivity have been reported and discussed elsewhere, with additional details about the study design and participants demographics [12, 40, 46]. All methods conform to the Code of Ethics by the World Medical Association (Declaration of Helsinki) and were approved by the Independent Research Ethics Committee, Uppsala University and the Medical Products Agency in Sweden. Informed consent was obtained from all subjects. EU Clinical Trial Register (https://www.clinicaltrialsregister.eu), number: 2008–003123–24.

**Table 1:** Demographic data for placebo (*n* = 17) and combined oral contraceptives (COC, *n* = 17) groups.

Demographic data	Placebo (*n* = 17)	COC (*n* = 17)
Age (years)	24.5 ± 3.3	25.5 ± 5.0
Menstrual cycle length (days)	27.3 ± 2.7	28.4 ± 2.3
Education (years)	15.6 ± 1.6	15.0 ± 2.3
Self-reported premenstrual syndrome symptoms, *n* (%)	9 (52.9%)	5 (29.4%)
Total duration of previous COC use (years)	4.4 ± 2.8	4.3 ± 3.0
**Mean difference in CD scores**^a^ **the third week of treatment and the third week of the pre-treatment cycle**		
Depressed mood	0.4 ± 0.9	1.0 ± 1.7*
Mood swings	0.1 ± 1.1	1.5 ± 1.3*
Irritability	0.2 ± 0.9	0.5 ± 1.8
Anxious, worried	0.03 ± 0.8	0.06 ± 1.1
Difficulties concentrating	0.05 ± 0.9	0.2 ± 1.1
Fatigue	0.2 ± 1.2	0.7 ± 1.3*
Disordered sleep	−0.05 ± 0.8	0.6 ± 1.2
Out of control feelings	0.2 ± 1.2	0.5 ± 1.2
**Hormonal values during the MRI-session**		
Estradiol pre-treatment (pg/ml)	173.35 ± 97.73	242.59 ± 174.93
Estradiol during-treatment (pg/ml)	435.65 ± 277.28**	36.76 ± 25.50**
Progesterone pre-treatment (ng/ml)	1.99 ± 0.91	3.07 ± 4.23
Progesterone during-treatment (ng/ml)	18.24 ± 21.18**	1.46 ± 0.57**

^*^Significantly greater than corresponding week during the pre-treatment cycle, *p* < 0.05–0.01. **Significantly higher for the placebo group, while lower for the COC group, *p* < 0.01.

^a^Each item of the CD score is a Likert scale, which ranges from 0 (absence of symptom) to 8 (maximal intensity).

### Experimental design

**Fig. 1:**
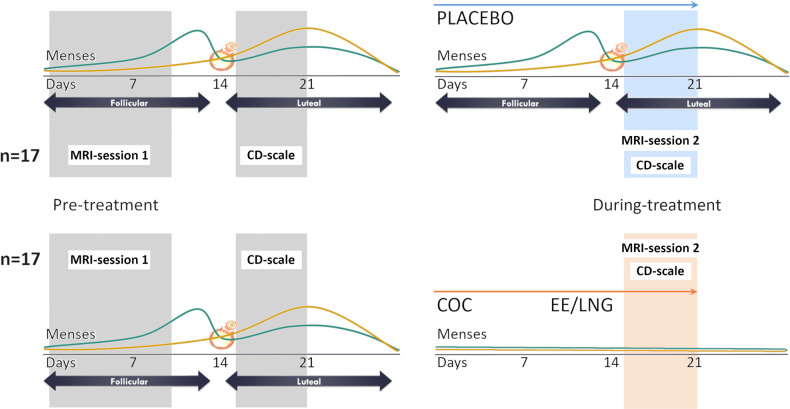
Experimental design. Each participant had two sessions, before and during treatment (day 1–10 and day 15–21 respectively after onset of menses). Therefore, for the placebo group endogenous hormone levels increased from the first to the second appointment, while in the COC group synthetic hormone levels were stable and endogenous hormones low. Expected hormonal variation in a physiological menstrual cycle and during COC treatment is represented by green (estradiol), and yellow (progesterone) lines. Actual values for each of the MRI-session are displayed in Table 1.

Participants were scanned twice, first during a pre-treatment cycle (day 4 ± 3 after onset of menses) and secondly, during the last week of the treatment cycle (day 15–21 after start of treatment). The participants started taking the pill on the first day of menses (Fig. 1). In the COC group serum concentrations of ethinyl estradiol and levonorgestrel were expected to be stable during treatment, with low levels of endogenous hormones. However, at the second scan, the placebo group was expected to have an increase in endogenous hormone levels (which, for most individuals, coincided with the luteal phase). Blood samples were drawn at each of the scanning sessions for hormonal analyses. After centrifugation and storage at −70 °C, samples were analyzed using a Roche Cobas e601 and Cobas Elecsys reagent kits (Roche Diagnostics, Bromma, Sweden). In order to confirm COC vs. placebo effect, hormonal levels between and within treatment groups were assessed. Given that hormone levels can differ from person to person, linear mixed models controlling for participants number as a random factor and the interaction between session and group were performed. For both estradiol and progesterone levels (Table 1) an interactive effect of group by session was found (estradiol: *b* = −2.11, SEb = 0.37, t(64) = −5.64, *p* < 0.001; progesterone: *b* = −1.41, SEb = 0.41, t(64) = −3.41, *p* = 0.001). As expected, while hormone levels increased in the placebo group from the first to the second MRI-session, in the COC group hormone levels decreased during treatment.

Additionally, participants filled out daily mood and physical symptoms on the Cyclicity Diagnoser (CD) scale [47] both during the pre-treatment and treatment cycles. This scale is composed, among others, by nine negative mood parameters that were analysed to assess mood deterioration: depression, interest in usual activities, fatigue, irritability, anxious/worried, mood swings, sense of being out of control, difficulties in concentrating and disordered sleep. Each item consists of a Likert scale from 0 (absence of the symptom) to 8 (maximal intensity of the symptom. COC-induced mood deterioration was defined as i) significant differences from the 21 pre-treatment days to the 21 treatment days, ii) 100% increase from baseline to the last week of the treatment cycle in summed negative mood symptoms, and iii) mean summed symptom scores higher than 9.0 during this last week [12]. As reported in previous analysis [12], COC users increased their scores of three emotional symptoms: *mood lability*, *fatigue* and *depressed mood*, while the placebo group showed no significant differences (Table 1). Given that some of the women reported having PMS-like symptoms, in this study we operationalized the changes pre- and during treatment as the standardized difference between the last week of the treatment compared to the corresponding pre-treatment week, in order to avoid confounding effects.

### Data acquisition

Functional and structural images were acquired on a Philips Achieva 3.0 T scanner using an 8-channel head coil (Philips Medical Systems, Best, The Netherlands). For the 5 min resting state a single shot echo planar imaging sequence was used to collect 100 volumes of BOLD data with a voxel size of 3.0 × 3.0 × 3.0 mm^3^ in 30 ascending slices (TR = 3000 ms, TE = 35 ms, flip angle = 90°, and FOV = 230 × 230 mm^2^). Participants were instructed to stay as still as possible and simply rest with their eyes closed. For the structural images an inversion recovery turbo spin echo sequence was used to acquire a structural T1-weighted image with a voxel size of 0.8 × 1.0 × 2.0 mm^3^ in 60 slices (TR = 5700 ms, TI = 400 ms, TE = 15 ms, and FOV = 230 × 230 mm^2^).

### Preprocessing

Scanner DICOM images were first converted to NIfTI files with MRIcron (www.nitrc.org/projects/mricron/). Images were pre-processed using SPM12 standard procedures and templates SPM12 (www.fil.ion.ucl.ac.uk/spm) and despiked using 3D-despiking as implemented in AFNI (afni.nimh.nih.gov). Pre-processing included realignment of the functional images, segmentation of the structural images, co-registration of the functional images to the structural images, normalization of functional images using the normalization parameters and spatial smoothing using a 6 mm kernel. The resulting images were subjected to the ICA-AROMA algorithm implemented in fsl including non-aggressive removal of artifactual components [48].

### Selection and extraction of volumes of interest

Eleven ROIs were selected as core nodes of the corresponding networks, based on a large body of literature. First, for the DMN, the precuneus/posterior cingulate cortex (PCC), bilateral angular gyri (AG) and medial prefrontal cortex (mPFC) [49, 50]. Second, for the SN, bilateral anterior insula (AI) and dorsal anterior cingulate cortex (dACC) [29, 50]. Third, for the ECN, bilateral middle frontal gyri (MFG) and supramarginal gyri (SMG) [51]. ROI specific masks were created with the Wake Forest University (WFU) Pickatlas toolbox [52]. Group-level peaks for each ROI were identified within each intrinsic connectivity network (ICN), using spatial ICA as implemented in the Group ICA for fMRI Toolbox (GIFT, http://mialab.mrn.org/software/gift) [53]. For this, we first extracted 20 components, identified each of them via spatial correlation to pre-existing templates [54], and selected those four corresponding to the DMN, SN, left ECN and right ECN. Functional connectivity between the ROIs was further corroborated to be positive between ROIs of the same network and anti-correlated between different networks using the CONN toolbox [55]. The ROIs, their functional connectivity and their group-level peak coordinates are listed in Table 2 and shown in Fig. S1 (supplementary material). In order to extract the principal eigenvariate from each of the 11 ROIs, subject-specific coordinates were determined as local maximum within 8 mm of the group-level coordinates, but restricted to still be within the ROI specific mask. The time series from each ROI were then used in subsequent DCM analyses.

**Table 2:** Group level coordinates for each region of interest.

Network	Region	MNI coordinates		
		X	Y	Z
**DMN**	**PCC**	0	−55	16
	**lAG**	−42	−63	28
	**rAG**	42	−65	28
	**mPFC**	0	60	−9
**SN**	**lAI**	−39	14	−2
	**rAI**	39	17	−3
	**dACC**	0	26	28
**ECN**	**lSMG**	−45	−49	45
	**rSMG**	46	−47	43
	**lMFG**	−39	26	32
	**rMFG**	38	26	34

*l* Left, *r* Right, *PCC* Precuneus/posterior cingulate cortex, *AG* Angular gyrus, *mPFC* Medial prefrontal cortex, *DMN* Default mode network, *AI* Anterior insula, *dACC* Dorsal anterior cingulate cortex, *SN* Salience network, *MFG* Middle frontal gyrus, *SMG* Supramarginal gyrus, *ECN* Executive control network.

### Spectral dynamic causal modeling and parametric empirical bayes

Resting state functional images were modelled using a (Bayesian) hierarchical random effects framework and spectral DCM was specified and inverted using DCM12 as implemented in SPM12 (www.fil.ion.ucl.ac.uk/spm). At the first level, for each participant and MRI-session, a fully connected model of 121 parameters (including all possible connections between nodes) was specified to fit the complex cross-spectral density estimating the intrinsic effective connectivity (i.e., the ‘A-matrix’) within and between networks. This estimation takes into account the effects of neurovascular fluctuations as well as noise [56]. Default priors implemented in SPM were used at this level and quality of the process was ensured by a variance explained by the model higher than 90% [38].

In order to compare changes in the treatment group to the changes observed in the placebo group, we ran a 3-level hierarchical analysis using a Parametric Empirical Bayes (PEB)-of-PEBs approach. We first modelled the treatment effect on each group separately, and then fit those parameters to the next level of the hierarchy (Fig. S1) as implemented in the Parametric Empirical Bayes framework in the SPM software [38, 44]. Specifically, we first modelled separate PEBs for each group, that captured either the effect of treatment on the placebo (PEB_1_) or the COC group (PEB_2_). Then the parameters of these two models were taken up to the third level of the hierarchy in a final PEB (PEB_3_). In this way, we captured the overall mean connectivity, the main effects of group (placebo vs. COC), and treatment (pre vs. during), and relevant for our research question, the interaction between group and treatment in PEB_3_ (Fig. S2. supplementary material). This hierarchical approach, with a general linear model (GLM) to capture effects of interest in the second and third level, allowed us to captured more accurately the between subjects’ variability (random effects).

In order to relate effective connectivity to mood symptoms during treatment, we modelled another PEB including the changes in CD-scale ratings that differed significantly between the COC and placebo group, i.e., *mood lability*, *fatigue* and *depressed mood*^1^ [12] (Fig. S3. supplementary material).

PEB results were thresholded to only include parameters from the A matrix that had a 95% posterior probability of being present vs. absent, which represents strong evidence for treatment-related changes, and thresholded to an estimated value Ep > 0.10. Only results surviving this threshold are reported in the results section.

### Cross-validation

In order to check whether the mood side effect related effective connectivity could predict the assignment of participants to one group or another (COC vs. placebo) we used a leave-one-out scheme (spm_dcm_loo.m) as described in [37]. This way we can assess the association between the actual group in the left-out-subject’s design matrix (pre-post COC-placebo) and the predicted group correspondence based on the left-out-subject’s connectivity. Given that there is a strong dilution-of-evidence effect, we selected those parameters that maximize the predictive accuracy based on their i) effect size and ii) relation to mood lability. Accordingly, we first thresholded the group by treatment interaction to Ep > 0.20 and then included only those parameters that were positively related to mood lability when increased during COC treatment and negatively related to this side effect when decreased during COC treatment.

## Results

The main effects of the COC and placebo treatment, and the interactive effect between group and treatment are displayed in Fig. S2 and summarized in Table. 3. Given that our research question focused on the connectivity changes in the COC treatment compared to the control group, only the interactive effects that surpassed a posterior probability of 95% and an estimated value of 0.10, will be described in the next paragraphs.

**Table 3:** Summary of connections that showed interactive effect of group by treatment.

		Group		Side effects^a^		
	Group*treatment	COC	Placebo	Mood lability	Fatigue	Depressed mood
From PCC to right AG	+	+		+	+	
From left AI to right AG	+	+		-		
From dACC to left SMG	+	+		+	+	-
From right AG to PCC	+	+			-	+
From right AG to left SMG	+	+		+	-	
From right AG to right SMG	+	+		+	-	
From left AG to right MFG	+	+		+	+	
From left AG to right AI	+	+		-		+
From left AG to mPFC	+	+	-	+		
**From dACC to PCC**	**+**	**+**	**-**	**+**		**-**
From dACC to mPFC	+	+	-	+	-	
From left SMG to PCC	+	+	-	-		
**From right MFG to dACC**	**+**	**+**	**-**	**+**		**+**
**From right AG to dACC**	**-**	**-**		**-**	**-**	
From right SMG to left MFG	-	-		-		+
From left MFG to right AG	-	-		-		+
From left MFG to PCC	-	-		-	-	+
From right MFG to right SMG	-	-	+	+		
From right AI to mPFC	-	-	+	-	+	-
dACC to itself	-	-	+	-		
From right AG to mPFC	-		+		-	+
From right AG to left AI	-		+	-	-	-
**From right AG to right AI**	**-**		**+**	**-**	**-**	**-**
From left AI to right AI	-		+		+	
From right AI to PCC	-		+	-		-
From left MFG to left AG	-		+	-		+
From left MFG to mPFC	-		+	-	-	-
From right MFG to left AG	-		+	-	-	
From right MFG to right AG	-		+		+	-
From right SMG to right MFG	-		+			
From left SMG to left AG	+		-	+		
From left SMG to right AG	+		-	-	-	+
From right SMG to left AI	+		-		-	
From right SMG to right AI	+		-			-
From left MFG to left SMG	+		-			
From PCC to rAI	+	-	- -	+		

All connections survived a 95% posterior probability threshold and an estimated value of 0.10. Columns 3–4 correspond to connectivity changes present during COC or placebo treatment respectively, indicated by a + if the connectivity increased and - if the connectivity decreased. Side effects modulation of the treatment connectivity are detailed in columns 5–7, indicated by a + if relationship to the connectivity parameter was positive, and - if the relationship was negative. Connections that were able to predict individual treatment are marked in bold.

^a^Relationship between during treatment connectivity and scaled difference between the last week of the treatment compared to the correspondent pre-treatment week.

**Fig. 2:**
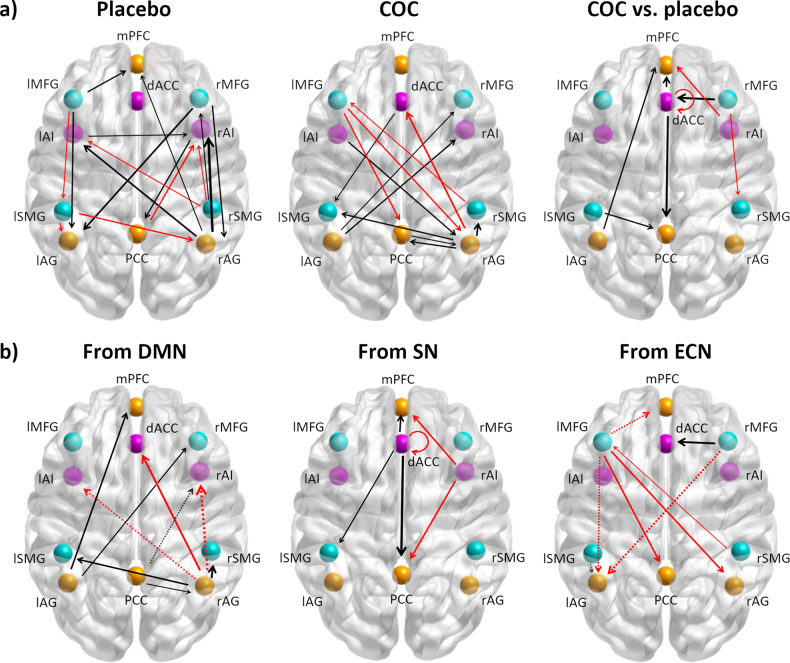
Interactive effects of group by treatment and relation to mood lability differences. **a** Interactive effects of group by treatment. These connections correspond to changes in the COC group during treatment compared to the placebo group if they surpassed a posterior probability of 95% and an estimated value of 0.10. The results are organized by changes observed in the placebo group, in the COC group, or both COC vs. placebo group in opposite direction. **b** Connections showing relationship to mood lability differences from DMN, from the SN, and from ECN. These connections surpassed a posterior probability of 95% and an estimated value of 0.10 and related during treatment to the mood-lability changes from the previous cycle. Only those changes in the same direction as the interactive effect of group by treatment are displayed. Dashed connections were those driven by the change only in the placebo group from follicular to luteal phase. For (**a**) and (**b**): The differential connectivity strengths (Ep) are depicted by the width of the arrow. Black arrows reflect positive values and red arrows reflect negative values.

Given that the placebo group connectivity was also subject to change due to the endogenous hormonal fluctuations of the natural menstrual cycle, we considered whether these interactive effects were due to changes (i) only in the placebo group, (ii) only in the COC group or (iii) in opposite direction for the COC and the placebo group. Results according to this classification are also illustrated in Fig. 2a. and summarized in Table 3.

### Changes only in the placebo group

From the follicular to the luteal phase, within-network connectivity increased in the DMN (from the right AG to the mPFC), and in the SN (from the left AI to the right AI). Within the ECN, we found a lateralized pattern: while connectivity increased from parietal to frontal ECN in the right hemisphere, connectivity decreased from frontal to parietal ECN in the left hemisphere.

Regarding the between-networks changes, we observed an increased connectivity between the DMN and the SN (from the right AG to bilateral AI), and in turn, from the right AI to the PCC. While frontal ECN increased its connectivity to the DMN (from the left MFG to the mPFC and to the left AG; and from the right MFG to bilateral AG), parietal ECN decreased its connectivity to the DMN (from the left SMG to bilateral AG). Connectivity from parietal ECN to SN also decreased (from the right SMG to bilateral AI).

### Changes only in the COC group

From pre- to during treatment MRI-session, within-network connectivity increased in the DMN (from the PCC to the right AG and viceversa), while it decreased in the ECN (from the right SMG to the left MFG). No significant changes were observed in the within-network connectivity of the SN.

Regarding the between-networks changes, we observed an increased connectivity between the DMN and the SN, with increased connectivity from the left AG to the right AI, and, in turn, increased connectivity from the left AI to the right AG. Between the DMN and the ECN several connectivity changes were observed during treatment. Connectivity increased from the left AG to the right MFG, and from the right AG to bilateral parietal ECN. In turn, the left MFG decreased its connectivity to the DMN (right AG and PCC). An increased connectivity between the SN and ECN (from the dACC to the left SMG) was observed, during treatment.

### Changes in opposite direction for COC and the placebo group

In the following, an increase refers to increased connectivity in the COC, but decreased connectivity in the placebo group; while a decrease refers to decreased connectivity in the COC, but increased connectivity in the placebo group.

Regarding the within-networks changes, we observed an increased connectivity in the DMN (from the left AG to the mPFC); whereas in the SN, the dACC decreased its self-connectivity, and in the ECN, connectivity from the frontal to the parietal right hemisphere also decreased.

Regarding the between-networks connectivity, most of the changes occurred between the DMN and the SN. While connectivity decreased from the right AI to the mPFC, it increased from the dACC to the medial nodes of the DMN (PCC and mPFC). In turn, the right AG decreased its connectivity to the dACC. Connectivity was increased from parietal ECN to posterior DMN (from the left SMG to the PCC), and from the frontal ECN to the medial SN (from the right MFG to the dACC).

### Summary of findings

Taking into account all the above, in the COC group compared to the placebo group, the within-network connectivity increased during treatment in the DMN, whereas it decreased in the SN and ECN. Regarding the between-network connectivity, specifically from the dACC (SN) to medial nodes of DMN, effective connectivity was increased in the COC group compared to the placebo group. From the rAG (posterior DMN) to the SN, effective connectivity increased in the placebo group compared to the COC group. Conversely, effective connectivity increased from the rAG to the posterior ECN in the COC group compared to the placebo group. Effective connectivity from the frontal ECN to the DMN was in general stronger during treatment in the placebo than in the COC group, while those connections originating in the lSMG followed the opposite pattern. In general, effective connectivity between the ECN and the SN was stronger in the COC group compared to the placebo group.

### Associations of effective connectivity mood side effects

When making the previous distinction among the connectivity changes, some consistent patterns could be distinguished in the relation to the side effects (see Table 3). Connections increasing in the COC group, independently of whether they also decreased in the placebo group or not, were in general positively related to mood lability. Connections decreasing in the COC group, independently of whether they also increased in the placebo group or not, were in general negatively related to mood lability. For those connections increasing only in the placebo group, we found mostly negative relationship to mood lability, while for those connections decreasing only in the placebo group, no consistent pattern of association to mood side effects was found (Table 3, Fig. 2b).

Among those mood lability-related connections, the following connectivity changes surpassed the threshold of Ep > 0.20: from dACC to PCC, from rMFG to dACC, from rAG to rAI, from rAG to dACC, and from rAG to rSMG. In order to maximize the predictive accuracy, only these connections were selected in the subsequent cross-validation analysis.

### Prediction of treatment by mood-related effective connectivity

**Fig. 3:**
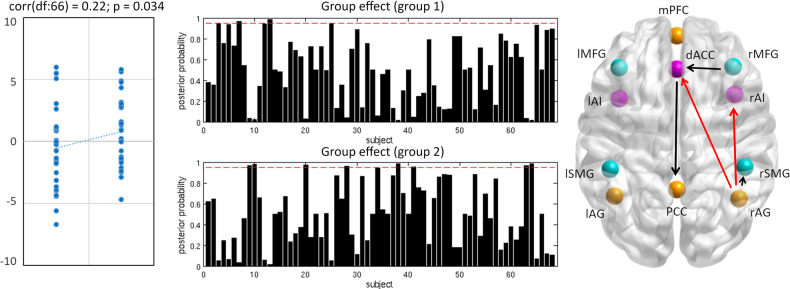
Leave-one-out cross-validation analysis. Left: scatter plot displaying the correlation between the actual treatment group in the left-out-subject’s design matrix and the predicted treatment group based on the left-out-subject’s connectivity. Centre: the resulting posterior probability for each treatment group for each subject. Right: Differential connectivity strength for the interactive effect of group by treatment. The differential connectivity strengths (Ep) are depicted by the width of the arrow. Black arrows reflect positive values and red arrows reflect negative values.

The leave-one out cross-validation based on those connections identified above as showing the highest effect size and relation to mood lability (dACC → PCC, rMFG → dACC, rAG → rAI, rAG → dACC, rAG → rSMG) showed a significant association between the actual and predicted group of *r*_df:66_ = 0.22, *p* = 0.03 (Fig. 3).

## Discussion

The main goal of the current manuscript was to characterize the changes in directed connectivity during COC treatment related to concurrent mood symptoms. Our results showed how effective connectivity changes noted during COC treatment related to mood deterioration. Mood lability was the most prominent COC-induced symptom [12], and also arose as the side effect most consistently related to connectivity changes. Connections that were related to increased mood lability showed increased connectivity during COC treatment, while connections that were related to decreased mood lability showed decreased connectivity during COC treatment. Among these, the connections with the highest effect size could also predict the participants’ treatment group above chance. Following the conceptual idea and analysis, we here also interpret the results within the triple network model framework, and in terms of connectivity changes within and between networks.

Overall, we found the expected pattern of enhanced connectivity within the DMN and decreased connectivity within the ECN during COC use [31, 57]. Increased connectivity within the DMN has been related to ruminative thoughts and lower connectivity within the ECN to lower cognitive control (see reviews [57, 58]. Specifically for the posterior DMN, increased within-network connectivity has been observed in major depression patients [59], suggested to underlie ruminative thoughts in this clinical population [58], and been related to premenstrual-like symptoms in COC users [22]. Interestingly, after antidepressant treatment, decreased functional connectivity within the posterior sub-network of the DMN, comparable to healthy subjects, has been reported [59].

However, and contrary to our hypothesis, only the connectivity from rAG to PCC was positively related to depressive symptoms. In that respect, different sub-items in depressive scales have been shown to be associated with abnormal activity of various brain areas [60]. On the other hand, an aberrant DMN as a neuropathological marker of major depression is still an open discussion and subject of controversy, due to small effect sizes, heterogeneity and high variability across patient samples [61]. The extent to which the depressive-like symptoms derived from this short time COC use and how they could relate to mood disturbances associated to longer intake duration, is still in question.

Alongside the decreased ECN within-network connectivity, which is line with previous findings [21], we observed a clear distinction in ECN-DMN between-network connectivity dynamics along the anterior-posterior axis. While connectivity from the left frontal ECN to the DMN decreased in COC-users, there was a bidirectional increase in connectivity between the posterior DMN (pDMN) and parietal ECN (pECN). The frontal nodes of the ECN are key regions for emotional processing and regulation [62, 63], whereas the parietal areas of the ECN and DMN appear to be crucial for the cognitive reappraisal of negative stimuli (see [64] for a meta-analysis). Disrupted activity of the frontal ECN has been observed for depression, anxiety, and bipolar disorder [31, 65–67]. Engagement of the parietal areas in mood and anxiety disorders has been proposed as a compensatory mechanism in response to impaired function of frontal areas [64]. Moreover, the frontal ECN has been suggested as a neural marker of clinical responsivity to treatment [68] and has been used as a target during antidepressant treatment with transcranial magnetic stimulation [69]. In the present sample, these connectivity changes were in general associated with mood lability but not consistently with depressive symptoms.

Remarkably, an important role seems to emerge for the dACC. Better emotional regulation and lower anxiety levels have been related to stronger dACC activity [70, 71], and depressive symptoms are associated with reduced dACC volume [72]. More importantly, this area has been identified as neural predictor of individual therapy response in depression [68] and anxiety disorders [73, 74]. In the present sample, the dACC seems to act as a mediator between the DMN and ECN. During COC-treatment, the medial nodes of the DMN are recruited by the dACC (compare also 40), while the dACC in turn is recruited by the frontal ECN. The over-recruitment of the ACC by the medial DMN has been described in major depressive [57] and anxiety disorders [31] in relation to impaired attention to relevant stimuli [19]. In healthy adults resting-state functional connectivity between PCC and ACC was positively related to both negative and positive daily mood and interpreted as a general enhanced reactivity to arousal as opposed to valence [75]. Regarding the engagement of the dACC to the frontal ECN, MFG-ACC hyper-connectivity has been suggested to reflect an increase in top-down control [76] as a compensatory mechanism in anxiety and depressive disorders [31]. It is worth noting that the pivotal role of the dACC seems also important for the group treatment prediction in the present sample.

The above-described changes and related experienced side effects could be a consequence of the synthetic hormones, the abolishment of cyclic endogenous hormonal fluctuations, or both. Although animal research shows a differential binding affinity of synthetic compared to endogenous hormones (levonorgestrel has a 5-fold affinity vs. progesterone for progesterone receptors) [3], some neuroactive effects of progesterone are mediated by its metabolites, i.e. allopregnanolone is a positive allosteric modulator of the GABA-A receptor, with anxiolytic properties [77]. Relatedly, long term contraceptive treatment in rodents (4–6 weeks including levonorgestrel) has shown to reduce cerebral progesterone and allopregnanolone and increase anxiety-like behaviour, related to changes in the GABAergic system [78, 79]. Different effects of androgenic vs. anti-androgenic progestins may not only depend on their interaction with the androgen receptor, but also their modulation of gluco- and mineralocorticoid receptors [80]. Each synthetic progestin present specific agonist or antagonist binding affinity for these receptors, which are crucial for the regulation of the stress response [81]. Moreover, low endogenous estradiol levels during COC use have been previously related to impaired fear extinction in a cross-species experiment with female rats and women [14], while high estradiol levels during the peri-ovulatory phase in naturally cycling women have been related to an advantage for emotion processing [82]. Therefore, we find it likely that the constant enhanced synthetic progestins effects, alongside the absence of endogenous hormonal cyclicity and estradiol-progesterone interaction may be of high relevance for the mood side effects and related changes in connectivity here described.

Some potential limitations need to be noted. First, an exclusion criterion was the use of hormonal contraceptives within the previous two months. While it could be argued that this represents a short wash-out period, it should be noted that prior to randomization, every participant had regular menstrual cycles. Second, the pre-treatment mood assessment was obtained during the luteal phase, while the pre-treatment MRI session was performed during menses. Correspondingly, differences in mood lability, fatigue and depressed mood were operationalized as the difference between the third week of treatment and the luteal pre-treatment phase, in order to avoid potential confounding effects of menstrual-cycle related variations. Last, although five minutes of resting state scanner sequence may seem short, and statistical significance is higher with increased sequence length, longer scans have been associated with similar or even diminished reliability [83, 84]. Furthermore, it has been shown that with this number of time points, the root mean squared errors and the parameters estimates after pooling across subjects, are still robust [56, 85]. Although it is out of the scope of this study, connectivity changes experienced by the placebo group along their menstrual cycle in this study did not entirely match changes in healthy naturally cycling women reported in our previous work [36]. The main reason for these discrepancies may be differences in the time window of each appointment, but individual variability in functional responsivity to sex hormones could have also contributed [86]. While the present study deliberately included women who had previously reacted with mood-effects to COC, they were also allowed to report experiencing premenstrual syndrome (PMS), while this condition was an exclusion criterion in the previous study. Accordingly, some of these differences could relate to both the vulnerability in women for PMS and the susceptibility to adverse mood effects during COC use.

In summary, the present randomized placebo-controlled trial showed effective connectivity changes during COC treatment related to worsened mood in women with a history of mood COC side effects. The most confident effects corresponded to connections that changed during COC treatment compared to placebo and were related to an increased in mood lability. These differences during COC treatment in the triple network model may affect cognitive processes important for mood stability and mental health and similar disruptions have been reported across mood disorders [19, 31]. Further studies are needed in order to shed light on specific mechanisms by which synthetic hormones exert changes on these neural substrates on one hand, and which specific features correspond to an increased vulnerability to experience adverse mood side effects on the other. The paucity of research regarding the neuroactive effects of COC calls for more extensive research, especially when considering the time window when women start using COC, due to the plasticity in adolescent brains.

## Supplementary information

Supplementary material

## Footnotes

1 Please note that the pre-treatment CD ratings were obtained during the luteal phase, while the pre-treatment MRI was performed during menses. This time-point was chosen to avoid potential menstrual-cycle related variations in the control group, while making sure that the changes in the COC group capture only those increases in mood lability, fatigue and depressed mood that surpass potential menstrual cycle related variations.
