# Supplementary material for: Triple network model of brain connectivity changes related to adverse mood effects in an oral contraceptive placebo-controlled trial
Source: Transl Psychiatry. 2023 Jun 16;13:209. doi: 10.1038/s41398-023-02470-x (PMC10276024; doi:10.1038/s41398-023-02470-x)
Supplement: Supplementary file 1 — Supplementary material [file 41398_2023_2470_MOESM1_ESM.docx]

**SUPPLEMENTARY MATERIAL**


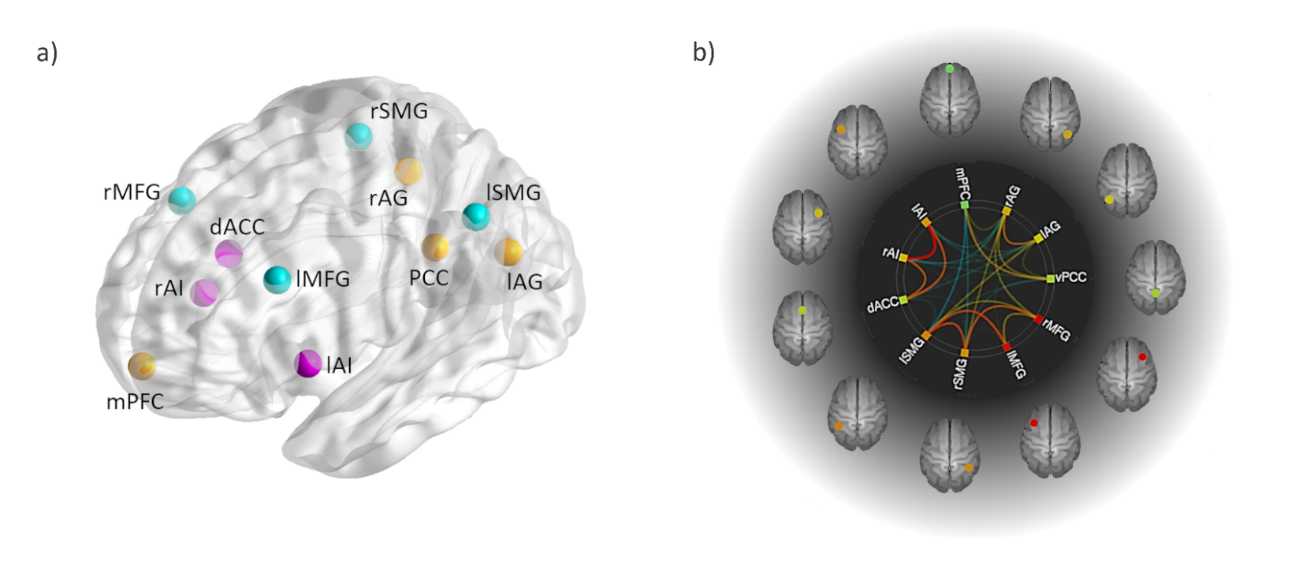

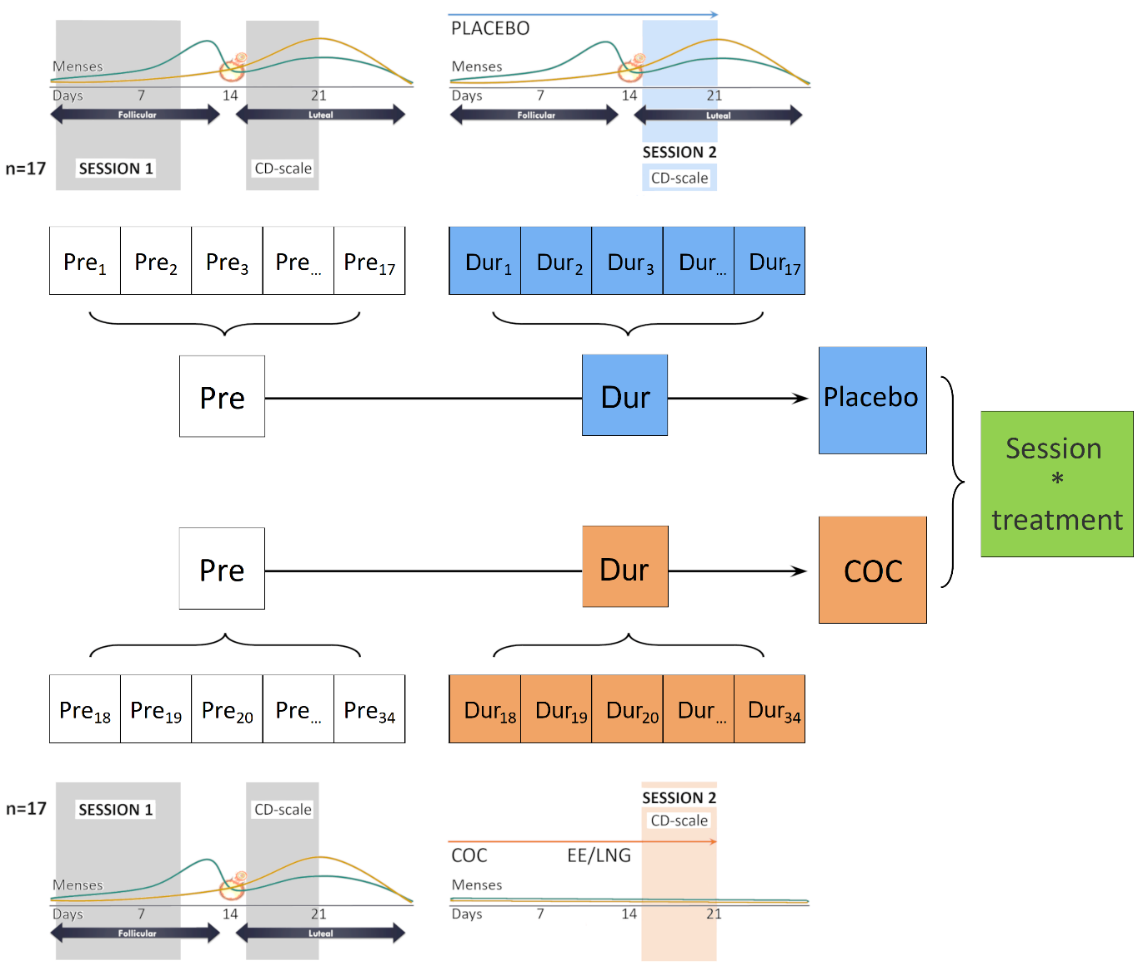


c)

**Fig. S1. Experimental design and analysis.** Regions of interest (ROIs, a) were specified based on functional connectivity analysis (b). ROIs in the same colour belong to the same intrinsic connectivity networks: in blue, executive control network; in yellow, default mode network; and in purple, salience network. b) Intrinsic connectivity networks were assessed by independent component analysis, and showed high positive (warm colours) within-network functional connectivity, while low positive, or negative (cold colours) connectivity to ROIs belonging to the other networks. c) 3-level hierarchical analysis design, Parametric Empirical Bayes (PEB)-of-PEBs. We first modelled each session (pre vs. during) and group (placebo in blue and COC in orange) separately, and then fit those parameters to the next level of the hierarchy: main effect of placebo and main effect of COC. In the third level, in order to capture interactive effects between treatment groups by session (pre and during treatment). Pre: pre-treatment; Dur: during treatment. PCC: precuneus/posterior cingulate cortex; AG: angular gyrus; mPFC: medial prefrontal cortex; AI: anterior insula; dACC: dorsal anterior cingulate cortex; MFG: middle frontal gyrus; SMG: supramarginal gyrus; l: left; r: right.


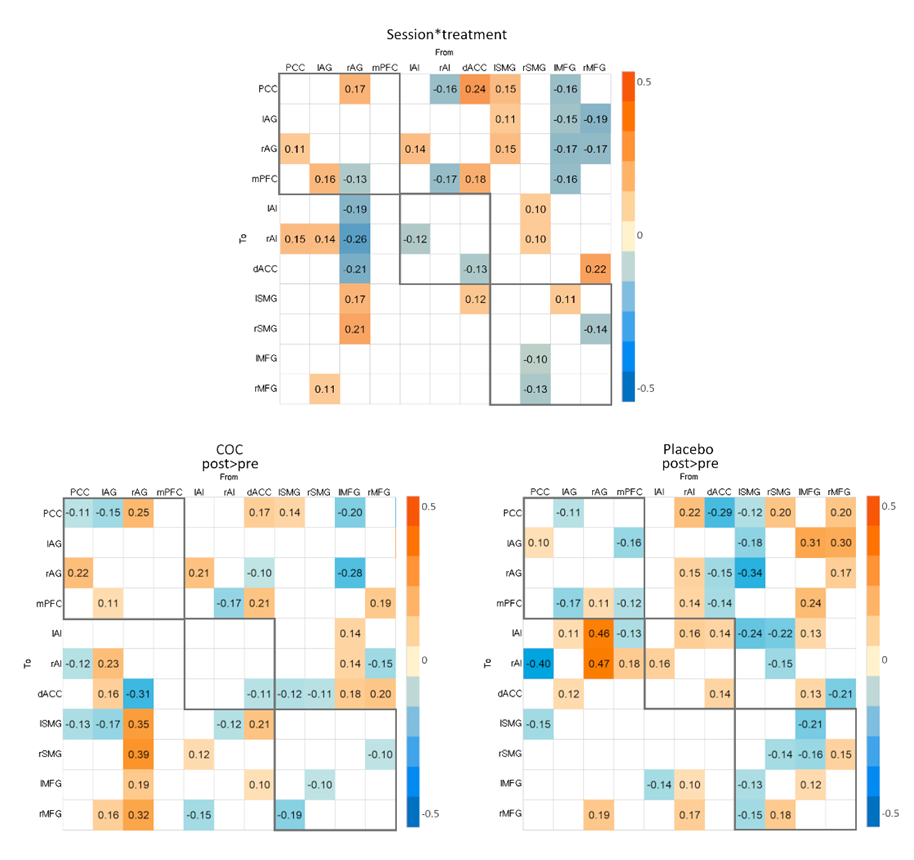


**Fig. S2. Estimated parameters for a) the interactive effect of session*treatment, b) COC treatment effect, c) placebo effect.** These connections surpassed a posterior probability of 95% and an estimated value (Ep) of 0.10. The exact Ep is indicated in each cell, warm colours indicating positive parameter estimates and cold colours negative. The columns are the outgoing connections, and the rows are the incoming connections, ordered as: PCC, lAG, rAG, mPFC, lAI, rAI, dACC, lSMG, rSMG, lMFG, and rMFG. PCC: precuneus/posterior cingulate cortex; AG: angular gyrus; mPFC: medial prefrontal cortex; AI: anterior insula; dACC: dorsal anterior cingulate cortex; MFG: middle frontal gyrus; SMG: supramarginal gyrus; l: left; r: right.

**
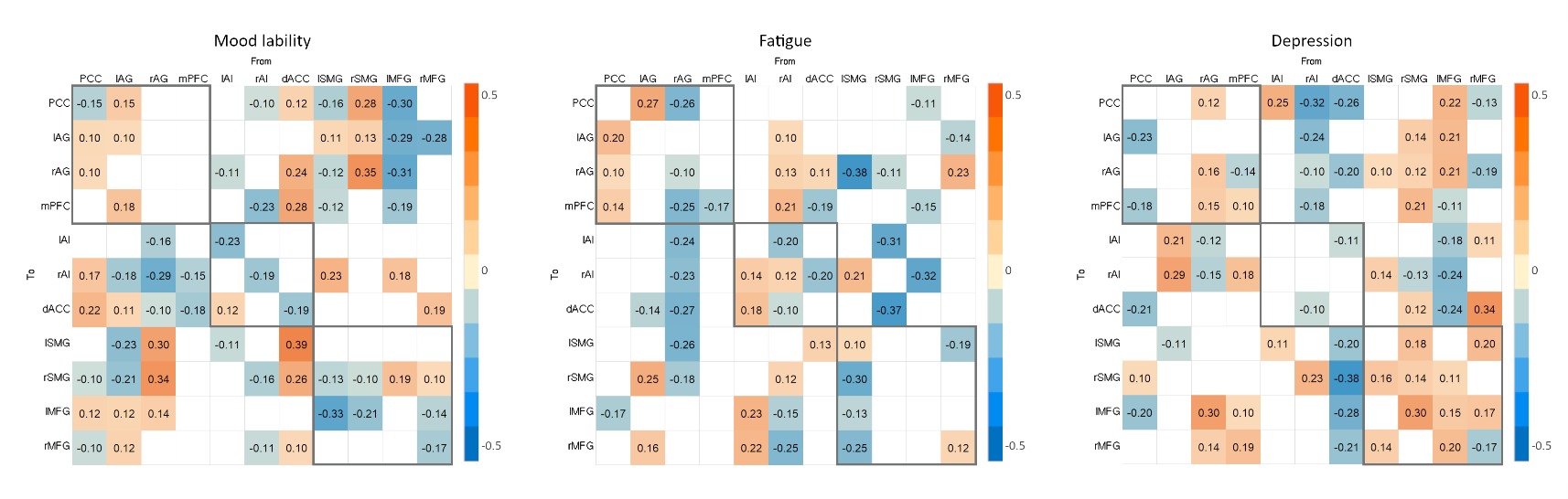
**

**
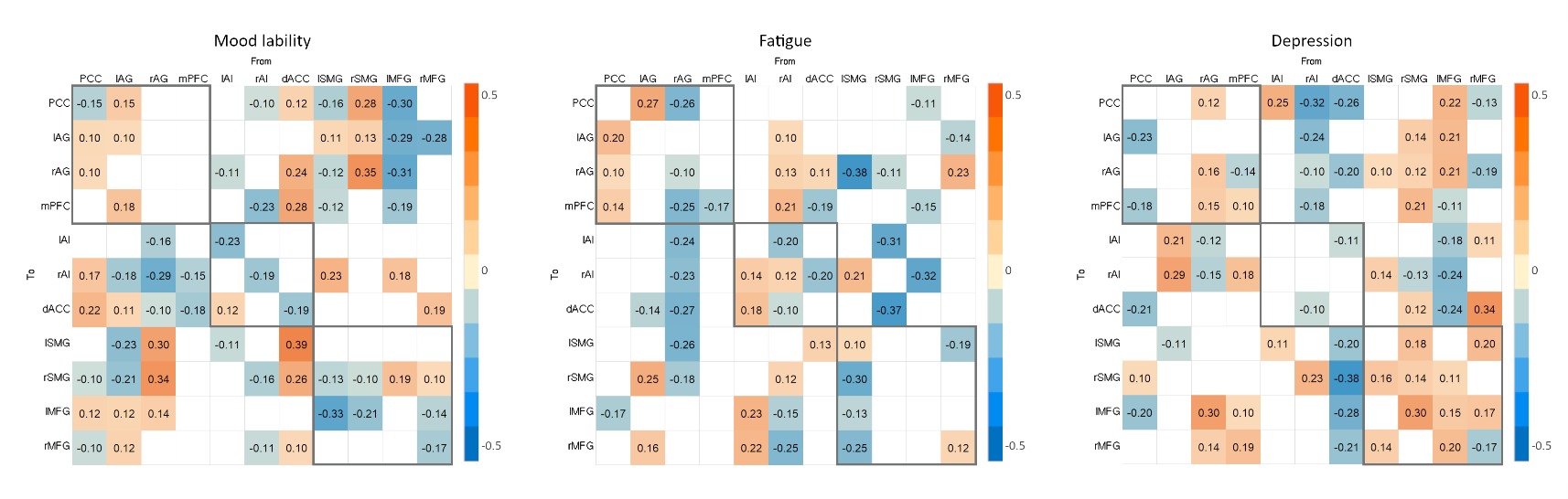

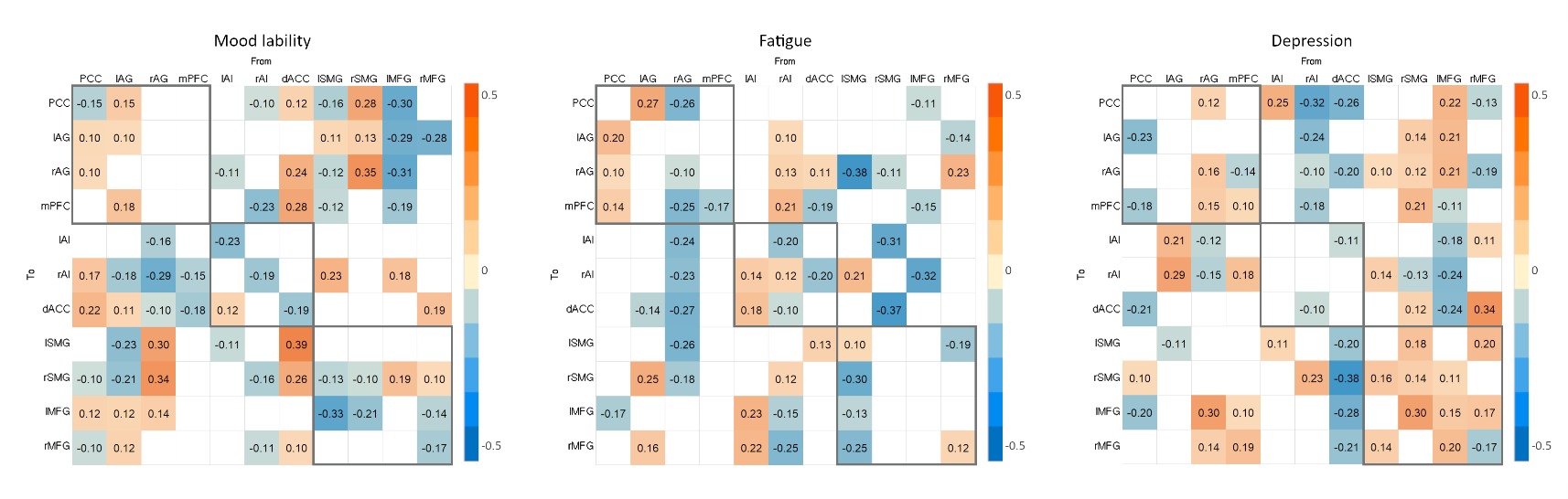
**

**Fig. S3. Estimated parameters for mood-related symptoms.** These connections surpassed a posterior probability of 95% and an estimated value (Ep) of 0.10. The exact Ep is indicated in each cell, warm colours indicating positive parameter estimates and cold colours negative. The columns are the outgoing connections, and the rows are the incoming connections, ordered as: PCC, lAG, rAG, mPFC, lAI, rAI, ACC, lSMG, rSMG, lMFG, and rMFG. PCC: precuneus/posterior cingulate cortex; AG: angular gyrus; mPFC: medial prefrontal cortex; AI: anterior insula; dACC: dorsal anterior cingulate cortex; MFG: middle frontal gyrus; SMG: supramarginal gyrus; l: left; r: right.
